# A Flow SPR Immunosensor Based on a Sandwich Direct Method

**DOI:** 10.3390/bios6020022

**Published:** 2016-05-13

**Authors:** Mauro Tomassetti, Giorgia Conta, Luigi Campanella, Gabriele Favero, Gabriella Sanzò, Franco Mazzei, Riccarda Antiochia

**Affiliations:** 1 Department of Chemistry, Sapienza University of Rome, P.le Aldo Moro 5, 00185 Rome, Italy; giorgia.conta@libero.it (G.C.); luigi.campanella@uniroma1.it (L.C.); 2 Department of Chemistry and Drug Technologies, Sapienza University of Rome, P.le Aldo Moro 5, 00185 Rome, Italy; gabriele.favero@uniroma1.it (G.F.); gabriella.sanzo@uniroma1.it (G.S.); franco.mazzei@uniroma1.it (F.M.); riccarda.antiochia@uniroma1.it (R.A.)

**Keywords:** ampicillin, flow immunosensor SPR, sandwich method

## Abstract

In this study, we report the development of an SPR (Surface Plasmon Resonance) immunosensor for the detection of ampicillin, operating under flow conditions. SPR sensors based on both direct (with the immobilization of the antibody) and competitive (with the immobilization of the antigen) methods did not allow the detection of ampicillin. Therefore, a sandwich-based sensor was developed which showed a good linear response towards ampicillin between 10^−3^ and 10^−1^ M, a measurement time of ≤20 min and a high selectivity both towards β-lactam antibiotics and antibiotics of different classes.

## 1. Introduction

β-lactams antibiotics are a class of antibiotics with a structure containing a β-lactam ring, *i.e.*, a four-term cyclic amide. The action mechanisms of most of these antibiotics interfere with the synthesis of the cell wall of the bacteria foreign to the human body [1]. Therefore, the β-lactam antibiotics generally act as bactericides. However, some bacteria are capable of producing particular enzymes, called β-lactamases, that are able to inactivate the same antibiotics, making them completely ineffective [1]. Among the antibiotics of the penicillin group, ampicillin shows a broader spectrum of activity than penicillin G. The cephalosporins, such as ceftriaxone (which will also be considered in this study), do not belong to the group of penicillins, but they belong to the same class of β-lactam antibiotics. Therefore, they have the same action mechanism of penicillins, although they show a wider antibacterial spectrum and a greater resistance to β-lactamase.

Of course, several strategies have been developed for the analytical determination of these antibiotics, for example chromatographic [2,3,4,5], mass-spectrometry [6,7,8,9], or microbial screening methods [10,11], but also methods based on sensors, biosensors and immunosensors [12,13,14,15,16,17,18,19]. Our research group developed different sensors for β-lactam antibiotics detection, initially ISES (Ion Selective Electrodes) [20] and most recently amperometric immunosensors [21].

Recent research carried out in our laboratories have been aimed at developing a traditional highly sensitive amperometric immunosensor [21] for penicillin G and other β-lactam antibiotics based on the competitive method, obtaining results that support the validity of this approach [21]. The LOD (low detection limit) was of the order of 10^−10^ M, but the analysis time was very long (about 1 h). This type of immunosensor also exhibited a poor selectivity towards other β-lactam antibiotics but a good selectivity towards antibiotics which do not belong to this class: it has therefore been used for the determination of the “pool” of antibiotics of the β-lactam class in common real matrices such as river waste water which can be more or less polluted by these types of antibiotics.

In the present research, we examined the possibility of developing an immunosensor for the direct determination of a β-lactam antibiotic, specifically ampicillin, by using the flow SPR technique, especially in order to shorten the analysis time. The SPR immunosensors are analytical devices capable of detecting, in a selective manner, chemical or biological species, which do not require sample pretreatments and the use of competitive methods.

However, the first results showed that it was not possible to realize an SPR immunosensor using the simplest and most common direct method, as the SPR signal obtained when, for instance, the penicillin G molecule that formed the immunocomplex with the antibody immobilized onto the SPR optical surface, was too low, probably because of the insufficiently high molecular weight of this antibiotic. In addition, the attempt to use a competitive method by immobilizing the antigen (instead of the antibody) and making it compete with the antigen to be determined in order to bind he free antibody in solution, was not successful, as described in this work. Moreover, this competitive method would not show any potential advantage compared to the traditional competitive method with amperometric detection in terms of measurement times, which would have been about the same for both methods. In fact, once the antibody complex is formed, its hydrolytic cleavage, in order to regenerate the optical surface of the SPR (that is the gold plate functionalized with the immobilized and not complexed antigen) to make a subsequent SPR measurement, resulted in being extremely difficult, as described in the “Results” section of the present paper. The research has therefore moved in the direction of the development of an immunosensor for ampicillin detection that is able to operate with a sandwich geometry (1st immobilized antigen molecule—antibody—2nd antigen molecule) that allowed for decreasing the analysis time and simplifying the measurement method.

## 2. Materials and Methods

### 2.1. Materials

Sodium dihydrogen phosphate NaH_2_PO_4_ (≥99.0%), sodium dibasic phosphate Na_2_HPO_4_ (≥99.0%), EtOH (96%), 11-mercaptoundecanoic acid (MUA) (95%), 1-ethyl-3-(3-dimethyl aminopropyl) carbodiimide (EDC), N-hydroxysuccinimide (NHS) (98%), glycine, ethanolamine (≥99%), ceftriaxone and erythromycin were supplied by Sigma-Aldrich (St. Louis, MO, USA). The SPR plates, composed of a layer of Au of a thickness of 50 nm on a glassy support, were supplied by XanTec bioanalytics GmbH (Dusseldorf, Germany). The oil with a refractive index of 1.6100 ± 0.0002 was supplied by Cargille Laboratories (Cedar Grove, NJ, USA). Sodium Ampicillin was provided by Farmitalia—Carlo Erba (Milan, Italy); anti-Ampicillin was provided by Sigma (St. Louis, MO, USA); penicillin G was produced by Fluka Analyticals (St. Louis, MO, USA); fosfomycin was produced by Crinos S.P.A. (Villa Guardia, Como, Italy).

All solutions were prepared with deionized water (*R* = 18.2 mΩ × cm at 25 °C; TOC (Total Organic Carbon) < 10 g·mL^−1^) obtained by a Millipore Milli-Q system (Millipore, Molsheim, France).

### 2.2. Apparatus

SPR flow measurements were realized by using BioSuplar 400T apparatus (Analytical μ-Systems—Department Of Mivitec GmbH, Sinzing, Germany), with a laser diode with low power (630–670 nm) as the light source. This instrument allows for the qualitative analysis of molecules on the basis of the mass of the sandwich antibody formed on the plate, which produces a variation in the resonance angle. This signal is then amplified and transduced. The output signal is successively processed as a function of time in the form of a sensorgram.

## 3. Results

In this section, we described all the steps carried out during the experimental work in order to develop a sandwich immunosensor for ampicillin detection and to construct the relative calibration curve.

### 3.1. SPR Immunosensor and Flow Measurements

An SPR immunosensor is an analytical device capable of selectively detecting chemical or biological species without the need of sample pretreatment. It is composed of three main elements: a bioreceptor (or biological component), a transducer, and finally a system for signal processing. The bioreceptor is immobilized on the surface of the optical sensor and is responsible for the variation of the resonance angle, the characteristic parameter of this technique, related to the species to be determined. In fact, we basically measure the variation of the resonance angle, due to the interaction between the analyte and the bioreceptor, in our case the antibody complex, immobilized on a suitably functionalized gold metal film. The “optical” signal generated is transduced into an electric signal, then amplified and processed in the form of a sensorgram (resonance angle *vs.* time).

**Figure 1:**
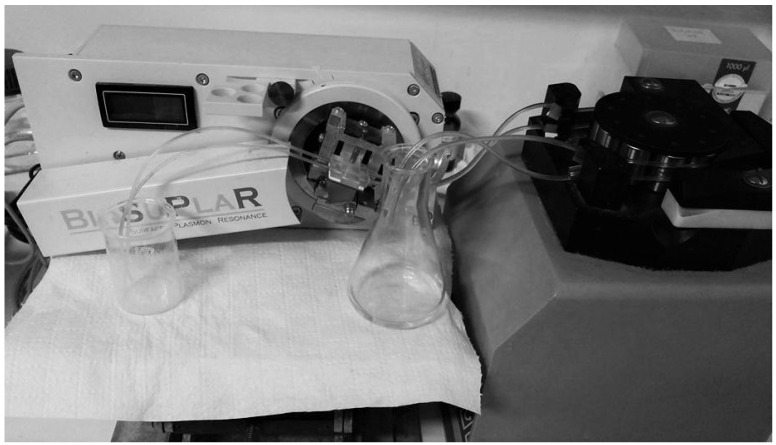
Measurement instrument used for Surface Plasmon Resonance (SPR) operating in flow mode.

**Figure 2:**
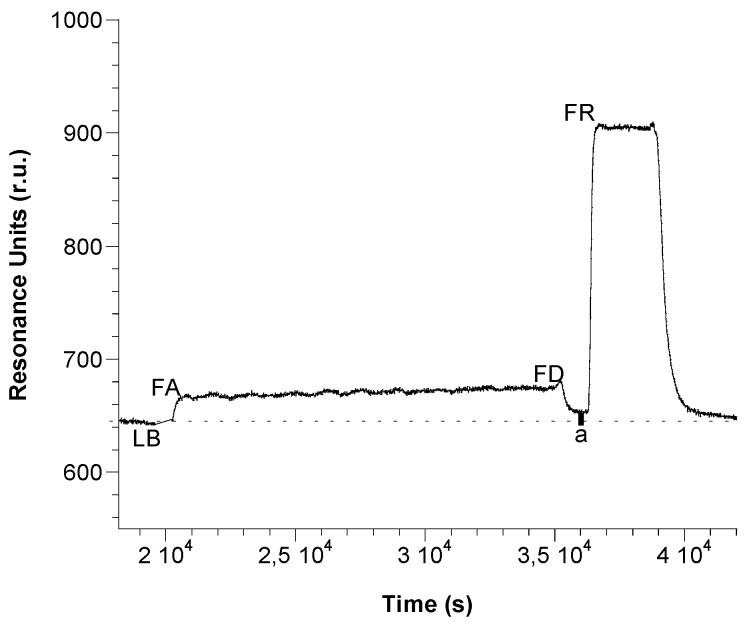
Example of sensorgram obtained with a sandwich format. For each measurement, after checking the original baseline (**LB**) by flowing phosphate buffer 50 mM, pH 7.2, the analyte that flowed into the system upon the steady state has been reached (**FA**); then, the dissociation step (**FD**) started by flowing again phosphate buffer (the small bold line (**a**) indicates the Δ r.u, relative to the formation of the immunocomplex between analyte and bioreceptor); the regeneration step (**FR**) was performed by glycine-HCl solution pH 2.4 inally the phosphate buffer to obtain the baseline.

The SPR flow device (Figure 1) has a prism as the main element, installed on a rotating plate, automatically controlled by a computer. The different samples are introduced into a proper cell by means of a peristaltic pump. The sensor consists of a gold sheet, 50 nm thick, placed on a glassy support. Once functionalized by forming a so-called SAM (Self-Assembled Monolayers), the plate is placed on the prism surface using an oil with a refractive index equal to that of the prism and the support itself, in order to ensure optical continuity. Finally, the plate is fixed with a system for mechanical anchorage. After positioning the SAM on the prism, the assembly of the SPR flow device is completed with a cell suitable for flow analysis. When the software for data processing is ready, it is possible to start the analysis. To observe the SPR phenomenon, the polarized light, emitted by a diode laser at low power, is reflected from the wafer on a detector and monitored as a function of the resonance angle, thanks to the prism rotation [22,23]. Successively, the SPR angle change caused by the formation of the sandwich was recorded, in the form of a sensorgram, as a function of time. The registered variation of resonance angle is proportional to the amount of complexed analyte. The results showed on the sensorgram have several phases (Figure 2) corresponding to the presence of different solutions in the flow cell. The SPR used unit is the Unit of Resonance (r.u.), which corresponds to a change of 0.0001° of the resonance angle.

### 3.2. Self Assembled Monolayer (SAM)

**Figure 3:**
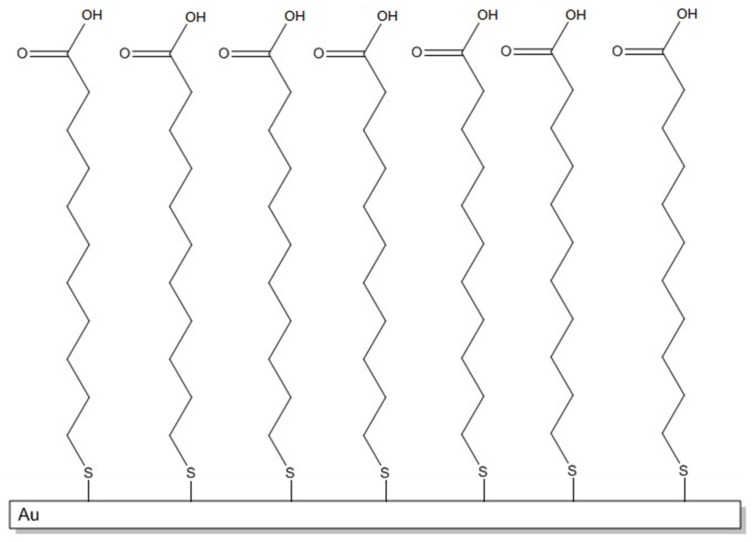
Schematic representation of SAM (Self Assembled Monolayer) with free –COOH and covalent bonds Au-S.

SAMs are highly ordered structures [24] that form spontaneously. A typical SAM is generally constituted by organosulfur compounds such as the 11-mercaptoundecanoic acid (MUA), which covalently binds to the gold surface of the plate. The preparation is carried out by dipping the plate in a solution of MUA 2 mM in ethanol for about 18 h. A SAM structure is therefore obtained, with the mercapto groups of the MUA able to form Au-S covalent bonds, while the carboxylic groups, which remain free, as shown in Figure 3, are available for subsequent binding with the antibody or the antigen. After 18 h, the plate with SAM is taken away from the solution, washed with ethanol and let to dry. The prism is rinsed with ethanol, then a drop of the oil is placed onto the prism surface and the prepared SAM-modified gold plate.

The stabilization of the SPR signal is then carried out by flowing 50 mM phosphate buffer into the cell at pH = 7.2 for about 1 h. This process is crucial as it rehydrates the MUA surface and creates a stable baseline on the sensorgram.

### 3.3. Choice of the Measurement Format

As mentioned in the Introduction and successively described in the Results and Discussion section, it was found that, after the immobilization of the antigen (ampicillin) on SAM and the association of its antibody (anti-ampicillin), a strong bond is formed, not easy to break through the hydrolysis with the classic eluent solutions normally used for this purpose [21,22,23]. This suggested the development of a sandwich geometry for the measurement instead of a competitive format type. After the injection of Gly-HCl at pH 2.4, the antibody complex, immobilized on the plate surface, and consisting of the first immobilized antigen—antibody—second antigen is in fact “regenerated” by breaking the bond with the second antigen. This suggests that the bond between the second antigen molecule and the antibody is certainly much weaker than that between the antibody and the first antigen molecule immobilized on the plate, which however is not easily broken by the treatment with the same solvent (Gly-HCl at pH 2.4).

### 3.4. Immobilization of Ampicillin

**Figure 4:**
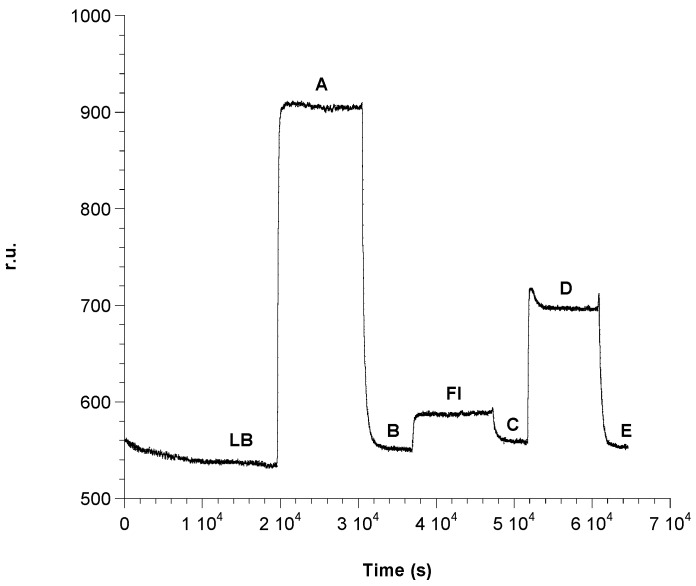
Immobilization of ampicillin: (**LB**) original baseline generated by flowing phosphate buffer; (**A**) activation with **EDC/NHS**; (**B**) washing with phosphate buffer; (**FI**) immobilization of ampicillin 10^−2^ M; (**C**) washing with phosphate buffer; (**D**) inactivation of –COOH groups which may have not reacted with ethanolamine; (**E**) washing with phosphate buffer.

**Figure 5:**
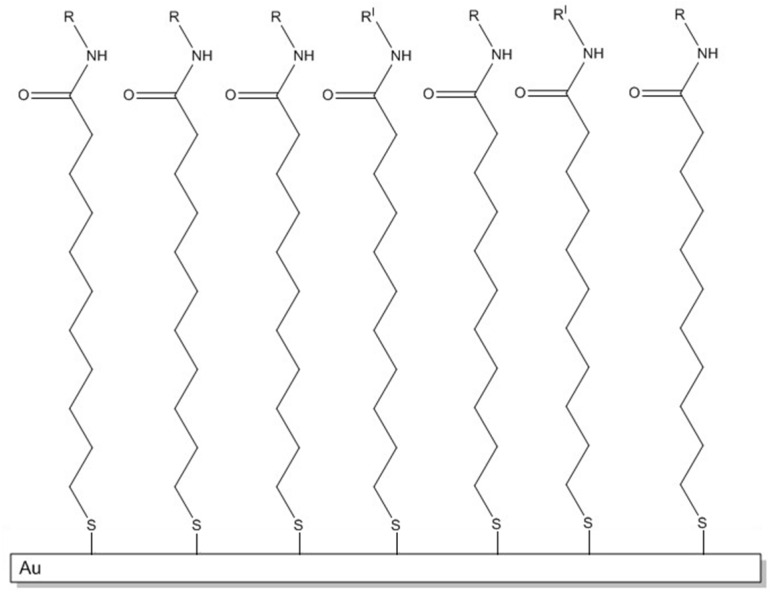
Schematic representation of SAM association with ampicillin 10^−2^ M (**R**) and inactivation of –COOH groups which may have not reacted by ethanolamine (**R’**).

The immobilization procedure was studied by monitoring the sensorgram reported in Figure 4. The first operation was the SAM stabilization, which occurred in the cell by the phosphate buffer flowing 50 mM at pH = 7.2 for about 1 h. This allowed for recording a first base line (LB line, Figure 4). Successively, a 1:1 mixture of 0.5 M EDC and 0.1 M NHS is allowed to flow in the cell for about 15 min in order to activate the carboxyl groups of the SAM, with a consequent raising of the signal (A line, Figure 4). The plate is then washed with 50 mM phosphate buffer, pH = 7.2, with a resulting lowering of the signal due to separation from the surface of the excess of reagents (B line, Figure 4). At this point, a solution of ampicillin 10^−2^ M is allowed to flow in the cell for about 40 min (FI line, Figure 4) in order to form the covalent bonds (amidic bonds) between the carboxy groups of the SAM and the antigen (Figure 5). Finally, the cell was washed with phosphate buffer (C line, Figure 4). In order to avoid non-specific interactions with the activated –COOH groups, the latter are blocked by ethanolamine flowing 1 mM (Figure 5) for about 15 min (D line, Figure 4. The cell was then washed with phosphate buffer (E line, Figure 4).

As shown in Figure 4, ampicillin was effectively immobilized on the functionalized surface. In fact, the base line (C), after the step of ampicillin immobilization (FI), is located at r.u. values greater than the base line (B) before the ampicillin immobilization.

### 3.5. Association of Anti-Ampicillin

**Figure 6:**
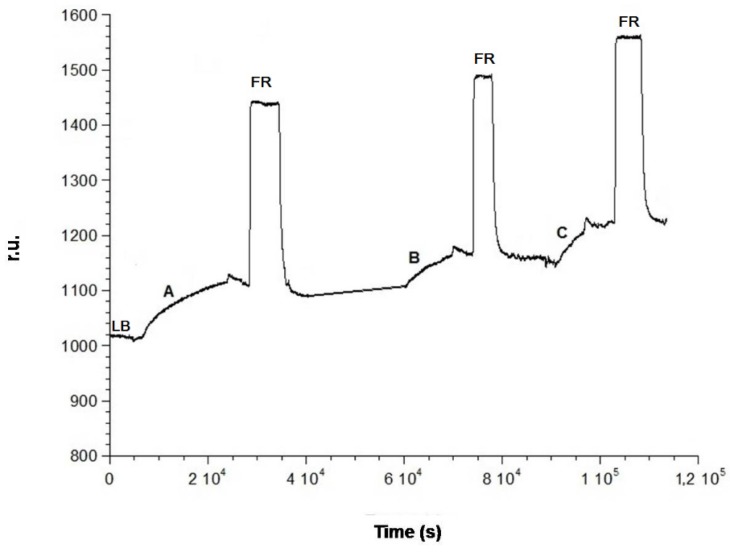
Antibody complexation on the SAM coated with the antygen: (**A**) 6 × 10^−10^ M; (**B**) 8 × 10^−10^ M; (**C**) 1 × 10^−9^ M, anti-ampicillin flowing successive solutions; (**FR**) regeneration step by flowing Gly-HCl at pH 2.4.

**Figure 7:**
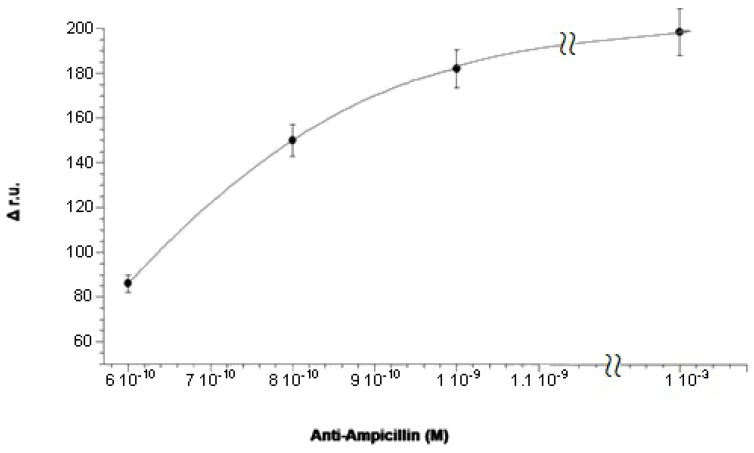
“Saturation curve” with anti-ampicillin, obtained by flowing antibody solutions: 6 × 10^−10^ M, 8 × 10^−10^ M, 1 × 10^−9^ M, 1 × 10^−3^ M).

The surface is now ready for the formation of the antibody complex. Subsequent injections of different solutions of antibody at increasing concentrations have been performed. These have been produced from a stock solution with a concentration of 1 × 10^−3^ M. The first step was to flow 50 mM of phosphate buffer at pH = 7.2 to obtain the base line (LB line, Figure 6). The first antibody solution, at a concentration of 6 × 10^−10^ M, was introduced into the cell observing the increase of the relative signal until a plateau is reached (A, Figure 6) due to the formation of the antibody complex. At this point, a washing procedure with phosphate buffer was carried out. Then, a solution of Gly-HCl, pH = 2.4 was introduced into the cell, which should, in theory, dissociate the complex between nti-ampicillin and ampicillin (FR line, Figure 6) through a hydrolysis reaction with the consequent regeneration of the activated surface, where only the immobilized antigen is present. The signal was expected to return to the initial reference level, before carrying out a second addition of the antibody. On the contrary, it is easy to see on the sensorgram that the complex between anti-ampicillin and ampicillin immobilized on the gold surface is practically not dissociated after the treatment with Gly-HCl, pH = 2.4. The surface not being regenerated, hence it is impossible to carry out a second measurement with the same plate, so that it would also be impossible to build, for example, a calibration curve using a “competitive” method with the same plate. The experiment was therefore continued with successive additions of further solutions of antibody at increasing concentrations, 8 × 10^−10^ M (B line, Figure 6) and 1 × 10^−9^ M (C line, Figure 6), until, for concentration above at least 10^−3^ M, almost all the immobilized antigen molecules had practically reacted with the antibody, by forming the antibody immobilized complex. The Δ r.u. values obtained were plotted against the concentration of anti-ampicillin, obtaining an antibody saturation curve, as shown in Figure 7. The obtained “saturation curve” allowed for evaluating the concentration of antibody solution capable of virtually saturating with the antibody the plate already activated with the antigen, by formation of the antibody complex, immobilized on the plate itself. This concentration is at least higher than 1 × 10^−3^ M, under the operating suitable conditions. It should be noted that the instrument was able to detect the antibody complex when the increase of its concentration was several orders of magnitude lower, since anti-ampicillin is a molecule with quite a high molecular weight (150 kDa).

### 3.6. Formation of the Sandwich and Construction of a Calibration Curve for Ampicillin

**Figure 8:**
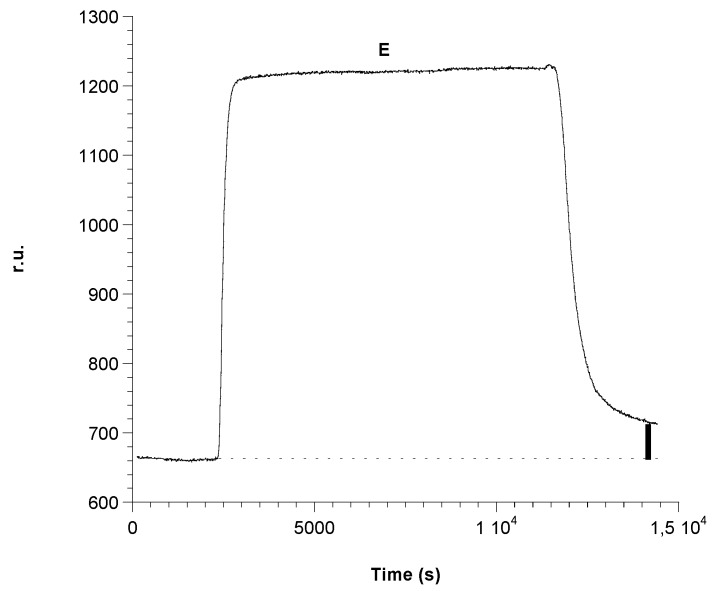
Example of ampicillin detection 1 × 10^−1^ M with sandwich format. Δ r.u (bold line) is related to the concentration (**E**) 1 × 10^−1^ M, before and after the association of ampicillin.

**Figure 9:**
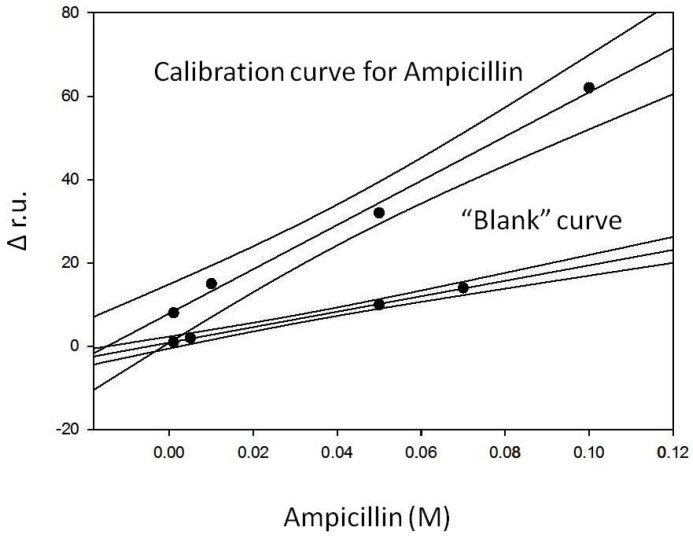
Comparison between blank curve and calibration curve of ampicillin.

The experiments described in the previous paragraph confirmed the difficulty with applying a competitive method and therefore suggested the choice to use the sandwich geometry to obtain a calibration curve for ampicillin. Therefore, after completely saturating the plate with a 0.1 M antibody solution and washing it with buffer, as described in the previous paragraph, the experience started by recording the initial baseline when 50 mM phosphate buffer at pH = 7.2 was introduced in the flow cell containing the plate activated with the antibody complex immobilized on its surface. Successively, several injections of solutions at increasing ampicillin concentration, obtained by diluting the same stock solution (1 × 10^−1^ M), have been carried out. The following parts of the curve were obtained: the flow of the first ampicillin solution at a concentration of 1 × 10^−3^ M on the antibody immobilized complex caused a small increase of the signal; once a stable signal is reached, phosphate buffer was again injected. A small decrease of the signal was observed, but at the end of the decrease, a positive difference signal (r.u.) compared to the original base line was registered, which proved the sandwich formation. The subsequent injection of a solution of Gly-HCl at pH 2.4 caused a characteristic increase of the signal. Lastly, by flowing again phosphate buffer 50 mM at pH = 7.2, the signal returned to a value of r.u. equal to that of the initial original base line (Figure 2). This showed that the sandwich had broken, but the antibody complex remained fixed on the plate. The same steps were repeated at increasing ampicillin concentrations from 5 × 10^−3^ M to 1 × 10^−1^ M. An increasing value for the difference between the signal observed after the washing with phosphate buffer and the initial original base line (example of Figure 8) has been recorded, which demonstrated how the interaction between the 2nd antigen molecule and the antibody was established each time giving rise to the sandwich geometry. A typical example of a recorded measurement for 1 × 10^−1^ M ampicillin concentration is reported in Figure 8. It was clear, however, that this observation was to be supported by a “blank” test, which is described in the following paragraph. In conclusion, in the described experience, the Δ in r.u. values related to each antigen addition have been determined and the difference, in terms of resonance units, between the final r.u. values (after washing with phosphate buffer), obtained with the association of the second antigen with the antibody and the r.u. values relative to the initial original baseline, was always positive and growing. The so obtained Δ in r.u. values were finally plotted as a function of the relative ampicillin concentration (see calibration line, Figure 9). For ampicillin concentrations lower than 10^−3^ M, it is likely that the sandwich is formed, but the sensitivity of the method does not seem enough to highlight it.

### 3.7. “Blank” Curve for Ampicillin

In order to make a “blank” curve, the usual SAM was prepared by dipping the gold plate for about 18 h in a solution of 2 mM 11-mercaptoundecanoic acid and ethanol. After the fixed time, the SAM was washed with ethanol to remove the excess reagent. Then, the plate was left to dry. The assembly was completed by placing on the prism a drop of oil and then the prepared plate with the SAM. Once obtained, the base line through the flow of phosphate buffer (50 mM at pH = 7.2) for about 1 h, the –COOH groups have been activated with a solution of EDC 0.5 M and NHS 0.1 M at a 1:1 ratio (freshly prepared) for about 15 min, then, the plate was washed with phosphate buffer and finally a solution of ampicillin 10^−1^ M was injected and allowed to flow for about 40 min. The plate was then washed again with 50 mM phosphate buffer at pH = 7.2. In order to be sure that all the sites on the gold plate had been saturated, a solution of 1 mM ethanolamine was injected to block any carboxyl groups that had not reacted. At this point, after removing the excess ethanolamine by washing with phosphate buffer, the following ampicillin solutions at increasing concentrations have been injected: (a) 10^−3^ M; (b) 5 × 10^−3^ M; (c) 5 × 10^−2^ M; (d) 7 × 10^−2^ M. The Δ in r.u. values, determined after each addition, were plotted as a function of increasing concentrations of ampicillin. The “blank” curve thus obtained and the calibration curve described in the previous section were both reported in a single graph (Figure 9) in which each point represents the average of three separate determinations.

The equations of the straight lines obtained and the main analytical data are summarized in Table 1. The fact that the two lines show very different slopes (the slope of the “blank” curve is very low) indicates that the sandwich geometry was actually formed and allowed the construction of the calibration curve, while the immobilization of the antigen alone on the SAM, as in the case of the “blank” curve, gave a signal which is almost zero and barely visible only at the highest antigen concentrations.

### 3.8. Selectivity Tests

In theory, the response of each immunosensor should be very selective because of the specificity of the antigen-antibody reaction. However, this selectivity should be experimentally verified due to the cross-reactivity, a situation in which it is possible that side reactions occur at the immunosensor due to a chemical compound very similar to the antigen. Consequently, the selectivity was assessed considering both compounds with a structure similar to ampicillin, such as other β-lactam antibiotics (penicillin G and ceftriaxone) and other antibiotics with very different structures (fosfomycin and erythromycin), as possible interferents.

**Figure 10:**
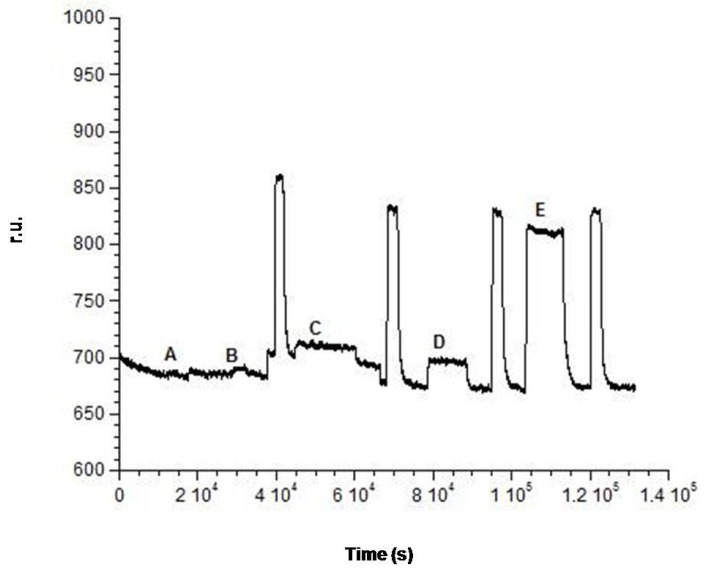
Selectivity: complexation of penicillin G by using sandwich immunosensor format. Penicillin G solutions: (**A**) 5 × 10^−4^ M; (**B**) 1 × 10^−3^ M; (**C**) 5 × 10^−3^ M; (**D**) 1 × 10^−2^ M; (**E**) 5 × 10^−2^ M.

**Figure 11:**
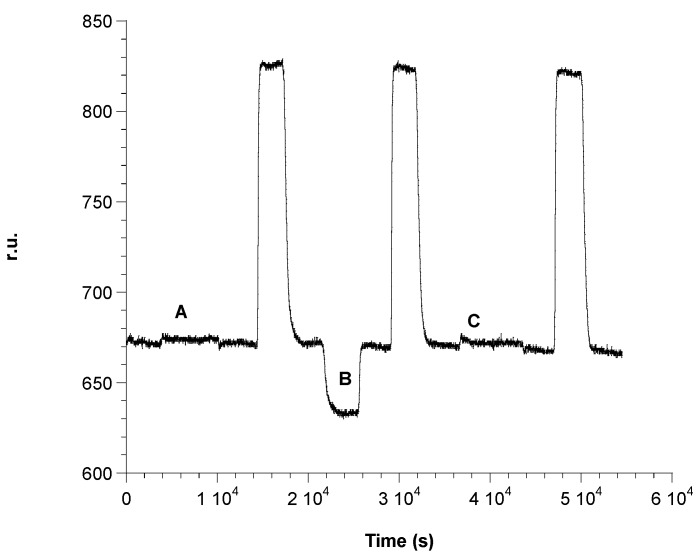
Selectivity test: (**A**) Fosfomycin 1 × 10^−3^ M; (**B**) Erythromicin 5 × 10^−3^ M; (**C**) Ceftriaxone 4 × 10^−3^ M.

The first selectivity test involved penicillin G (Figure 10), an antibiotic with the same β-lactam ring of ampicillin. Different concentrations of penicillin G have been tested, similar to those used to construct the calibration curve of ampicillin, and injected in succession in the cell as reported in Section 3.6, obtaining the sensorgram shown in Figure 10. It is possible to note that the instrument seems at first to detect the presence of Penicillin G in the cell but also that, after washing with 50 mM phosphate buffer at pH = 7.2, the signal returned to the original base line. This can be ascribed to the fact that the sandwich in the case of penicillin G may not form, or at least it is not detected by the SPR transducer, probably because of the low molecular weight of penicillin G. In other selectivity tests (Figure 11), possible interferences of both β-lactam, such as ceftriaxone, or cephalothin, and non β-lactam antibiotics, such as fosfomycin, or erythromicin, were evaluated by using the same procedure employed for penicillin G.

Also in the case of ceftriaxone, a cephalosporin, which is a β-lactam antibiotic but does not belong to the same class of ampicillin, the formation of the sandwich was not detected (line C of Figure 11). In the case of fosfomycin, the Δ in r.u. before and after the injection of this antibiotic, resulted in being almost zero (line A of Figure 11). Similar experiments have been carried out with erythromycin, another non β-lactam antibiotic. Its injection resulted in a decreasing signal (line B of Figure 11), as if this addition would cause a partial separation of the antibody complex. However, this assumption does not seem fully explanatory, as both fosfomycin and erythromycin showed Δ in r.u. values equal to zero after the subsequent injection of 50 mM phosphate buffer at pH = 7.2 with a return of the signal to the original baseline on the sensorgram. These results showing that the latter two antibiotics do not create any interference could perhaps be predictable, as they are non β-lactam antibiotics. Based on this evidence, it is possible to conclude that the immunosensor developed for ampicillin detection results in being very selective towards this antibiotic.

## 4. Conclusions

The purpose of this experimental research was the construction of an immunosensor for the direct determination of ampicillin, using the SPR technique operating in flow conditions. In order to reach the aim of the work, the results obtained suggested the development of a sandwich geometry (first molecule of immobilized antigen—antibody—second antigen molecule) as the best format. The formation of the sandwich was confirmed by comparing the slope value of the calibration curve of ampicillin with the slope of the “blank” curve, which resulted being very low. Furthermore, from the data resulting from the selectivity tests, it was found that none of the other tested antibiotics, both those belonging to very different classes of antibiotics and those structurally similar to the class of β-lactam, resulted in being potential interferents. This is a remarkable result, as it highlights the great selectivity of the SPR immunosensor for ampicillin in the concentration range from 10^−3^ M to 10^−1^ M. A disadvantage of the developed SPR immunosensor, compared to traditional “competitive” immunosensors (non-SPR), is that traditional immunosensors are generally more sensitive devices [21] compared to the SPR immunsensor described in this research, even if they have often proven to be much less selective compared to the SPR immunosensor. However, for certain applications such as the control of commercial pharmaceutical specialties, a high sensitivity is not mandatory. Moreover, good selectivity means that the method could be used in the case of specialties containing different active molecules in association.

Another important positive aspect to be emphasized is the analysis time. In fact, a direct determination of ampicillin in a much shorter time (maximum 20 min) compared to the time required by the traditional competitive methods (which generally require more than 1 h [21] to complete the measure) is possible, thanks to the formation of the sandwich.

## Figures and Tables

**Table 1:** Analytical data using an SPR device of both calibration and blank curves.

	Equation of the Regression Straight Line	Linearity Range (M)	*R*^2^	Pooled SD (%)	LOD (M)
Calibration straight line (Y = Δ r.u., X = M )	y = 7.89 (± 1.63) + 530.8 (± 29.1)x	1 × 10^−3^–1 × 10^−1^	0.9940	5.1	10^−3^
“Blank” curve (Y = Δ r.u., X = M)	y = 0.91 (± 0.15) + 185.3 (± 3.4)x	1 × 10^−3^–7 × 10^−2^	0.9993	4.4	10^−3^

## Abbreviations

The following abbreviations are used in this manuscript:

SPR	Surface Plasmon Resonance
LOD	Low detection limit
SAM	Self Assembled Monolayer
r.u.	Unit of Resonance
MUA	11-mercaptoundecanoic Acid
Gly	Glycine
EDC	1-ethyl-3-(3-dimethyl aminopropyl) Carbodiimide
NHS	N-hydroxysuccinimide

## References

[1] Murray P.R., Rosenthal K.S., Pfaller M.A. (2015). Microbiologia Medica.

[2] Benito-Peña E., Partal-Rodera A.I., Léon-González M.E., Moreno-Bondi M.C. (2006). Evaluation of mixed mode solid phase extraction cartridges for the preconcentration of beta-lactam antibiotics in wastewater using liquid chromatography with UV-DAD detection. Anal. Chim. Acta.

[3] Marazuela M.D., Bogialli S. (2009). A review of novel strategies of sample preparation for the determination of antibacterial residues in foodstuffs using liquid chromatography-based analytical methods. Anal. Chim. Acta.

[4] Briscoe S.E., McWhinney B.C., Lipman J., Roberts J.A., Ungerer J.P.J. (2012). A method for determining the free (unbound) concentration of ten beta-lactam antibiotics in human plasma using high performance liquid chromatography with ultraviolet detection. J. Chromatogr. B.

[5] Samanidou V.F., Evaggelopoulou E.N., Papadoyannis I.N. (2006). Development of a validated HPLC method for the determination of four penicillin antibiotics in pharmaceuticals and human biological fluids. J. Sep. Sci..

[6] Jin H., Kumar A.P., Paik D.H., Ha K.C., Yoo K.C., Lee Y.I. (2010). Trace analysis of tetracycline antibiotics in human urine using UPLC–QToF mass spectrometry. Microchem. J..

[7] Heller D.N., Smith M.L., Chiesa O.A. (2006). LC/MS/MS measurement of penicillin G in bovine plasma, urine, and biopsy samples taken from kidneys of standing animals. J. Chromatogr. B.

[8] Becker M., Zittlau E., Petz M. (2004). Residue analysis of 15 penicillins and cephalosporins in bovine muscle, kidney and milk by liquid chromatography–tandem mass spectrometry. Anal. Chim. Acta.

[9] Ohmori T., Suzuki A., Niwa T., Ushikoshi H., Shirai K., Yoshida S., Ogura S., Itoh Y. (2011). Simultaneous determination of eight β-lactam antibiotics in human serum by liquid chromatography–tandem mass spectrometry. J. Chromatogr. B.

[10] Pikkemaat M.G., Rapallini M.L., Dijk S.O., Elferink J.W. (2009). Comparison of three microbial screening methods for antibiotics using routine monitoring samples. Anal. Chim. Acta.

[11] Musser M.B., Anderson K.L., Rushing J.E., Moats W.A. (2001). Potential for milk containing penicillin G or amoxicillin to cause residues in calves. J. Dairy Sci..

[12] Brand U., Reinhardt B., Rüther F., Scheper T., Schügerl K. (1990). Bio-field-effect transistors as detectors in flow-injection analysis. Anal. Chim. Acta.

[13] Goldfinch M.J., Lowe C.R. (1984). Solid-phase optoelectronic sensors for biochemical analysis. Anal. Biochem..

[14] Adrian J., Pasche S., Voirin G., Adrian J., Pinacho D.G., Font H., Sánchez-Baeza F., Marco J.M.P., Diserens J.M., Granier B. (2009). Wavelength-interrogated optical biosensor for multi-analyte screening of sulfonamide, fluoroquinolone, β-lactam and tetracycline antibiotics in milk. Trends Anal. Chem..

[15] Thavarungkul P., Dawan S., Kanatharana P., Asawatreratanakul P. (2007). Detecting penicillin G in milk with impedimetric label-free immunosensor. Biosens. Bioelectron..

[16] Benito-Peña E., Moreno-Bondi M.C., Orellana G., Maquieira A., Van Amerongen A. (2005). Development of a novel and automated fluorescent immunoassay for the analysis of β-lactam antibiotics. J. Agric. Food Chem..

[17] Park E.K., Jung W.C., Lee H.J. (2010). Application of a solid-phase fluorescence immunoassay to determine amoxicillin residues in fish tissue. Acta Vet. Hung..

[18] Gamella M., Campuzano S., Conzuelo F., Esteban-Torres M., De Las Rivas A.B., Reviejo A.J., Muñoz R., Pingarrón J.M. (2013). An amperometric affinity penicillin-binding protein magnetosensor for the detection of β-lactam antibiotics in milk. Analyst.

[19] Huth S.P., Warholic P.S., Devou J.M., Chaney L.K., Clark G.H. (2002). Parallux™ beta-lactam: A capillary-based fluorescent immunoassay for the determination of penicillin-G, ampicillin, amoxicillin, cloxacillin, cephapirin, and ceftiofur in bovine milk. J. AOAC Int..

[20] Campanella L., Tomassetti M., Sbrilli R. (1986). Benzylpenicillinate liquid membrane ion-selective electrode: Preparation and application to a real matrix (Drug). Ann. Chim..

[21] Merola G., Martini E., Tomassetti M., Campanella L. (2014). New immunosensor for β-lactam antibiotics determination in river waste waters. Sens. Actuators B Chem..

[22] Tomassetti M., Martini E., Campanella L., Favero G., Sanzò G., Mazzei F. (2013). Lactoferrin determination using flow or batch immunosensor surface plasmon resonance: Comparison with amperometric and screen-printed immunosensor methods. Sens. Actuators B Chem..

[23] Tomassetti M., Martini E., Campanella L., Favero G., Sanzó G., Mazzei F. (2015). A new surface plasmon resonance immunosensor for Triazine pesticide determination in bovine milk: A comparison with conventional amperometric and screen-printed immunodevices. Sensors.

[24] Love J.C., Estroff L.A., Kriebel J.K., Nuzzo R.G., Whitesides G.M. (2005). Self-Assembled Monolayers of thiolates on metals as a form of nanotechnology. Chem. Rev..

